# Genomic and Physiological Characteristics of a Novel Nitrite-Oxidizing *Nitrospira* Strain Isolated From a Drinking Water Treatment Plant

**DOI:** 10.3389/fmicb.2020.545190

**Published:** 2020-09-15

**Authors:** Hirotsugu Fujitani, Kengo Momiuchi, Kento Ishii, Manami Nomachi, Shuta Kikuchi, Norisuke Ushiki, Yuji Sekiguchi, Satoshi Tsuneda

**Affiliations:** ^1^Biomedical Research Institute, National Institute of Advanced Industrial Science and Technology, Tsukuba, Japan; ^2^Research Organization for Nano & Life Innovation, Waseda University, Tokyo, Japan; ^3^Department of Life Science and Medical Bioscience, Waseda University, Tokyo, Japan

**Keywords:** ammonia, comammox, genome, isolation, kinetics, nitrite, *Nitrospira*, *Nitrosomonas*

## Abstract

Nitrite-oxidizing bacteria (NOB) catalyze the second step of nitrification, which is an important process of the biogeochemical nitrogen cycle and is exploited extensively as a biological nitrogen removal process. Members of the genus *Nitrospira* are often identified as the dominant NOB in a diverse range of natural and artificial environments. Additionally, a number of studies examining the distribution, abundance, and characterization of complete ammonia oxidation (comammox) *Nitrospira* support the ecological importance of the genus *Nitrospira*. However, niche differentiation between nitrite-oxidizing *Nitrospira* and comammox *Nitrospira* remains unknown due to a lack of pure cultures. In this study, we report the isolation, physiology, and genome of a novel nitrite-oxidizing *Nitrospira* strain isolated from a fixed-bed column at a drinking water treatment plant. Continuous feeding of ammonia led to the enrichment of *Nitrospira*-like cells, as well as members of ammonia-oxidizing genus *Nitrosomonas*. Subsequently, a microcolony sorting technique was used to isolate a novel nitrite-oxidizing *Nitrospira* strain. Sequences of strains showing the growth of microcolonies in microtiter plates were checked. Consequently, the most abundant operational taxonomic unit (OTU) exhibited high sequence similarity with *Nitrospira japonica* (98%) at the 16S rRNA gene level. The two other *Nitrospira* OTUs shared over 99% sequence similarities with *N. japonica* and *Nitrospira* sp. strain GC86. Only one strain identified as *Nitrospira* was successfully subcultivated and designated as *Nitrospira* sp. strain KM1 with high sequence similarity with *N. japonica* (98%). The half saturation constant for nitrite and the maximum nitrite oxidation rate of strain KM1 were orders of magnitude lower than the published data of other known *Nitrospira* strains; moreover, strain KM1 was more sensitive to free ammonia compared with previously isolated *Nitrospira* strains. Therefore, the new *Nitrospira* strain appears to be better adapted to oligotrophic environments compared with other known non-marine nitrite oxidizers. The complete genome of strain KM1 was 4,509,223 bp in length and contained 4,318 predicted coding sequences. Average nucleotide identities between strain KM1 and known cultured *Nitrospira* genome sequences are 76.7–78.4%, suggesting at least species-level novelty of the strain in the *Nitrospira* lineage II. These findings broaden knowledge of the ecophysiological diversity of nitrite-oxidizing *Nitrospira*.

## Introduction

Nitrification is a key process in the biogeochemical nitrogen cycle. Conventionally, this reaction takes place in two-steps: the first step is ammonia oxidation, which is carried out by ammonia-oxidizing archaea (AOA) and ammonia-oxidizing bacteria (AOB), and the second step is nitrite oxidation, which is carried out by the nitrite-oxidizing bacteria (NOB). Recently, it has been reported that a group of *Nitrospira* species that are conventionally recognized as NOB, carry out complete ammonia oxidation (comammox), transforming ammonia to nitrate (Daims et al., 2015; van Kessel et al., 2015). This surprising finding suggests the ecological importance of *Nitrospira* and indicates that our understanding about nitrification is still insufficient.

*Nitrospira* are chemolithoautotrophic bacteria and are categorized into six different lineages, of which lineage II exhibits a huge phylogenetic diversity and environmental distribution (Daims et al., 2001; Lebedeva et al., 2008, 2011). To date, many environmental sequences within the *Nitrospira* lineage II have been identified from different environments, such as wastewater treatment plants (Schramm et al., 1998; Maixner et al., 2006; Park and Noguera, 2008; Fujitani et al., 2013; Gruber-Dorninger et al., 2015), freshwater habitats (Nowka et al., 2015b), permafrost soils (Nowka et al., 2015b), volcanic grassland soils (Daebeler et al., 2014), and rhizosphere (Okabe et al., 2012; Caliz et al., 2015). Additionally, recent metagenomic analysis revealed the presence of comammox *Nitrospira* in activated sludge (Chao et al., 2016; Camejo et al., 2017), soils (Orellana et al., 2018; Shi et al., 2018), freshwater (Liu et al., 2020), and drinking water treatment plants (DWTPs) (Pinto et al., 2015; Wang et al., 2017).

In systems producing drinking water, nitrification plays a key role in decreasing monochloramine and producing nitrate in DWTPs (Wang et al., 2014). Culture-independent approaches revealed that nitrite-oxidizing *Nitrospira* were more abundant than *Nitrobacter* in DWTPs (Regan et al., 2003; Tatari et al., 2017). As nitrite concentration is suggested as an important environmental factor differentiates niches among NOB (Daims et al., 2016) and between lineages I and II *Nitrospira* (Maixner et al., 2006; Fujitani et al., 2013), an increase of nitrite concentration selected for *Nitrotoga* rather than *Nitrospira* on biofilm systems fed with tap water (Kinnunen et al., 2017). To examine niche differentiation within NOB, it is important to characterize pure strains kinetically (Nowka et al., 2015a; Ushiki et al., 2017; Kitzinger et al., 2018). In rapid sand filters flowing into DWTPs in Denmark, *Nitrospira* were found to be present in abundance as a core taxon based on metagenomic approaches (Gülay et al., 2016; Palomo et al., 2016). Further analysis demonstrated that comammox *Nitrospira* were more abundant than AOB based on *amoA* gene abundance (Fowler et al., 2018) and had metabolic versatility allowing niche-partitioning among ammonia oxidizers (Palomo et al., 2018). Consequently, ammonia oxidation by comammox *Nitrospira* was functionally demonstrated by DNA and RNA stable isotope probing (Gülay et al., 2019). Although these culture-independent approaches revealed the occurrence of *Nitrospira* in oligotrophic environments, little is known about the physiology and genome of a *Nitrospira* strain from DWTPs. Recently, *Nitrospira inopinata*, the only comammox pure culture, was isolated from microbial biofilm on the surface of a pipe and characterized kinetically (Kits et al., 2017). Surprisingly, this strain seemed to be adapted to oligotrophic environments with a high affinity for ammonia but low affinity for nitrite. This finding raised additional questions about niche differentiation among NOB and between comammox *Nitrospira* and nitrite-oxidizing *Nitrospira*. To answer these questions, isolation and characterization of novel *Nitrospira* species from oligotrophic environments is essential. Here, we report a novel nitrite-oxidizing *Nitrospira* strain that was isolated from a fixed-bed column present at a DWTP. Continuous feeding of ammonia to imitate the natural environment successfully led to the enrichment of diverse nitrifiers, including *Nitrospira* species belonging to lineage II and AOB. Subsequently, nitrite-oxidizing *Nitrospira* belonging to lineage II was purified based on a microcolony sorting technique. Finally, the morphology, physiology, kinetics, and genome of a novel *Nitrospira* strain were characterized.

## Materials and Methods

### Sample Source and Continuous Feeding Bioreactor

Zeolite as a water purification material was set up at the drinking water treatment plant. Zeolite has an ability to adsorb ammonium, creating a suitable habitat for nitrifiers. Zeolite was sampled as seed biomass to cultivate the nitrifiers in May 2010. To enrich nitrifiers, a continuous feeding bioreactor to supply substrate in low concentration was set up (Supplementary Figure S1). The volume of the bioreactor used was 1.1 L. Non-woven fabric materials were used as a biomass carrier. The inorganic medium was comprised of NH_4_Cl (0.0038–0.304 g L^–1^; 0.071–5.683 mM), NaCl (0.116 g L^–1^; 1.985 mM), MgSO_4_⋅7H_2_O (0.040 g L^–1^; 0.332 mM), CaCl_2_⋅2H_2_O (0.073 g L^–1^; 0.497 mM), KCl (0.038 g L^–1^; 0.510 mM), KH_2_PO_4_ (0.034 g L^–1^; 0.250 mM), and trace elements (1 mL). The composition of trace elements was the same as described in our previous research study (Abe et al., 2017). An aerobic culture condition was maintained at a pH of around 6.9 and incubated in the dark to avoid light inhibition. In the drinking water treatment plant, the influent ammonia concentration was below 1 mg-N L^–1^ (0.071 mM). Therefore, the influent ammonia concentration was also maintained at 1 mg-N L^–1^ in the primary culture. Flow rate was 4.4 L day^–1^. The hydraulic retention time was adjusted to around 6 h. Loading was also carried out at a rate of 4.4 mg-N L^–1^ day^–1^. During a 0–100 days period from the cultivation start, zeolites and non-woven fabric materials were placed in the continuous feeding bioreactor. On days 100–200, only zeolites were removed. After 200 days, influent ammonia concentration was increased gradually to reach up to 70 mg-N L^–1^ (5 mM) on day 350. During this period, ammonia concentration in the bioreactor was maintained below 1 mg-N L^–1^ (0.071 mM).

### Chemical Analysis of Nitrogen Compounds During Enrichment Process

During the enrichment process, chemical properties were assessed. Ammonia concentration was measured by performing the colorimetric analysis with indophenol (Kandeler and Gerber, 1988). Additionally, nitrite and nitrate concentrations were measured according to the protocol described in a previous study (Fujitani et al., 2014).

### Sorting of Nitrifiers’ Microcolonies, Pure Cultivation, and Purity Check

Nitrifiers’ microcolonies from the enrichment samples were separated using the cell sorter as described in previous research studies (Ushiki et al., 2013; Fujitani et al., 2014, 2015; Abe et al., 2017). The sorted microcolonies were incubated for 1–2 months in the 96 well microtiter plates containing inorganic medium as described previously. However, it is important to note that the medium contained both, 5 mg-N L^–1^ ammonia (NH_4_Cl; 0.36 mM) and 5 mg-N L^–1^ nitrite (NaNO_2_; 0.36 mM), and the pH was adjusted to 7.5. Incubation was performed at 23°C under dark and static conditions. Cell growth and identification were confirmed by fluorescence *in situ* hybridization (FISH) and 16S rRNA gene sequence analyses. Pure strains identified as *Nitrospira* were transferred into test tubes and Erlenmeyer flasks, and were supplemented with 10 mg-N L^–1^ of nitrite (0.71 mM) in the medium every month for further characterization. Likewise, pure strains identified as *Nitrosomonas* were sub-cultivated with 10 mg-N L^–1^ of ammonia (0.71 mM) in the medium. Purity checks were carried out by microscopic observations based on FISH analysis and by using the heterotrophic media. The strain KM1 was transferred to 200-fold diluted Luria-Bertani (BD, NJ, United States), Nutrient Broth (BD) and R2A [polypeptone 500 mg L^–1^, casamino acid 500 g L^–1^, soluble starch 500 mg L^–1^, yeast extract 500 mg L^–1^, sodium pyruvate 300 mg L^–1^, KH_2_PO_4_ 500 mg L^–1^ (3.67 mM), MgSO_4_⋅7H_2_O 50.0 mg L^–1^ (0.415 mM)] media in the form of agar plates and liquid cultures.

### Fluorescence *in situ* Hybridization (FISH)

Fluorescence *in situ* hybridization was performed according to a previously described standard protocol (Amann et al., 1990). Oligonucleotide probes were fluorescently labeled with the hydrophilic sulfoindocyanine dye (Cy3) (Fasmac Co. Ltd., Atsugi, Japan) and are listed (Supplementary Table S1). Observations were carried out according to a previous study (Fujitani et al., 2014).

### DNA Extraction, PCR, and Cloning

DNA was extracted from the enrichment samples according to the standard protocol of the ISOIL extraction kit (Nippon Gene, Tokyo, Japan). Fragments of 16S rRNA and *amoA* genes were amplified using the total DNA with the listed primer sets (Supplementary Table S1). The PCR mixtures and purification of PCR products were conducted based on previously described protocols (Fujitani et al., 2013). Purified PCR products were cloned according to the protocol provided in the Qiagen PCR cloning plus kit (QIAGEN). To identify 16S rRNA gene sequences of pure strains cultivated in 96 well microtiter plates, bacterial DNA was extracted by heating at 95°C. The 16S rRNA gene was amplified with the 27f/1492r primer set. The sequences of about 750 bases were obtained by using the primer 27f. The cloning of the comammox *Nitrospira amoA* genes was performed according to the protocol described in a previous study (Fujitani et al., 2020).

### Phylogenetic Analysis

The regions of sequences with low quality were trimmed, and the contaminated sequences were removed. The cloned sequences of 16S rRNA genes were grouped into operational taxonomic units (OTU) with a similarity threshold of 98.7%. The OTUs were classified using the SILVA SINA algorithm (Pruesse et al., 2012). In addition, the closest strain to each OTU was searched using the NCBI Web BLASTn program. The 16S rRNA gene sequences of AOB, NOB, and comammox *Nitrospira* were aligned using the ClustalW tool of the MEGAX software (Kumar et al., 2018). Maximum-likelihood trees were constructed in MEGAX using the Tamura-Nei model. Bootstrap values were calculated by performing the iteration 1,000 times. The phylogenetic analysis of comammox *Nitrospira amoA* gene was conducted according to a previously described protocol (Fujitani et al., 2020).

### Electron Microscopy

Morphology of a *Nitrospira* pure strain was observed by scanning electron microscopy (SEM) and transmission electron microscopy (TEM), according to a previous study (Fujitani et al., 2014).

### Physiological Activity Test to Determine Optimum Temperature and Generation Time

Mean nitrite oxidation rates (μM day^–1^) were calculated during a 3 days incubation period at 4, 13, 22, 26, 30, 34, and 38°C using the exponentially growing pure strain. We used a fresh medium containing less than 1 mM nitrite in the pre-incubation step. After the cells were incubated with the medium for a few days, we checked the cells were in the exponential growth phase. The oxidation rates were often measured to evaluate the optimum temperature of NOB pure cultures (Nowka et al., 2015b; Kitzinger et al., 2018). The cell number and the amount of oxidized nitrite showed a good positive correlation (Nowka et al., 2015a; Ushiki et al., 2017; Ishii et al., 2020). The culture experiment for determining the generation time (see below) showed that the isolated cells grew exponentially 2–4 days after nitrite addition. To investigate the generation time, the isolate was incubated at 28°C after replacing the supernatant in order to remove the nitrate and by-products by centrifugation. Nitrite and nitrate concentrations were measured using the ion chromatography with a TSKgel SuperIC-Anion HS (IC-2010; Tosoh, Tokyo, Japan) according to the protocol described in our previous studies (Fujitani et al., 2020; Ishii et al., 2020). Cell numbers were estimated by performing qPCR analysis of the *nxrB* gene as described in a previous study (Ushiki et al., 2017). The *nxrB* gene was amplified by using a specific primer set (Supplementary Table S1). The purified *nxrB* products at known concentrations were diluted to 10–10^8^ copies μL^–1^ to generate a standard curve. The PCR efficiency was 103% with an *R*^2^ value of 0.99. The thermal cycler program used was as follows: an initial denaturing step at 98°C for 2 min, followed by 40 cycles of denaturation at 98°C for 10 s, annealing at 52°C for 10 s, and extension at 68°C for 40 s. The estimated *nxrB* gene copy number was divided by two, because the *Nitrospira* strain possesses two copies of the *nxrB* gene. The average generation time was determined based on the change in the number of cells in the period between the confirmation of cell growth and the depletion of nitrite. The minimum generation time was also calculated using two time points from the log phase.

### Kinetic Parameters for Nitrite Oxidation

As described in previous studies (Ishii et al., 2017; Ushiki et al., 2017), the kinetic parameters were calculated from multiple nitrite oxidation rates using exponentially growing cells. The cells were harvested by centrifugation, and resuspended using fresh mineral nitrite medium. The cell suspensions in glass containers were incubated at room temperature with shaking until the available nitrite content was depleted. Every 5–30 min, aliquots of 50 μL of the samples were collected and heated at 95°C for 5 min to inactivate the *Nitrospira* cells. Nitrite concentrations were measured by a colorimetric method using the Griess reagent (Hewitt and Nicholas, 1964). The experimental data were fitted to the following Michaelis–Menten kinetic equation: *V* = (*V*_*max*_ [*S*])/(*K*_*m*_ + [*S*]). Here, *K*_*m*_ is the apparent half-saturation constant for nitrite (μM NO_2_^–^), *V*_*max*_ is the maximum nitrite oxidation rate (μmol NO_2_^–^/mg protein/h), *V* is the nitrite oxidation rate, and [*S*] is the nitrite concentration (μM NO_2_^–^). For protein measurements, the cells were lysed using 0.15 M NaOH solution at 90°C for 30 min. Protein concentrations were measured by using the BCA protein assay kit (Takara).

### Inhibition Effect With Free Ammonia

The inhibitory constant for free ammonia was determined upon amending the medium with 0–3.6 mM NH_4_Cl. The estimated half-saturation constant (*K*’_*m*_) values in the presence of free ammonia were fitted to the following inhibitory constant equation: *K*’_*m*_ = *K*_*m*_ (1 + [*I*]/*K*_*i*_). Here, *K*_*i*_ is the inhibitory constant for free ammonia (μM NO_2_^–^), and [*I*] is the free ammonia concentration (mg-NH_3_ L^–1^ and μM NH_3_), which was calculated by assessing the NH_4_Cl concentration, temperature, and pH (Anthonisen et al., 1976). The other materials and methods were in accordance with the procedures that are described above.

### Genome Reconstruction and Annotation

DNA was extracted from the purified strain KM1 according to the standard protocol of the NucleoSpin Tissue DNA extraction kit (Takara). DNA sequencing, assembly, and manual curation of the genomes of different strains were performed as previously described (Sekiguchi et al., 2015; Ushiki et al., 2018). Coding sequences (CDS) were automatically predicted and annotated by using the DFAST ver. 1.2.3 (Tanizawa et al., 2018) and a published *Nitrospira* genome sequence (Koch et al., 2015). Average nucleotide identities (ANI) were calculated using fastANI with the default settings (Jain et al., 2018). Orthologous genes were identified using the OrthoVenn 2 web service tool (Xu et al., 2019) with an *E*-value of 1e-05. Toxin-antitoxin modules were searched using the TAfinder with *E*-value of 1e-06 for BLAST and *E*-value of 1e-03 for HMMer (Xie et al., 2018).

## Results and Discussion

### Enrichment of Nitrifiers Using a Continuous Feeding Bioreactor

**FIGURE 1:**
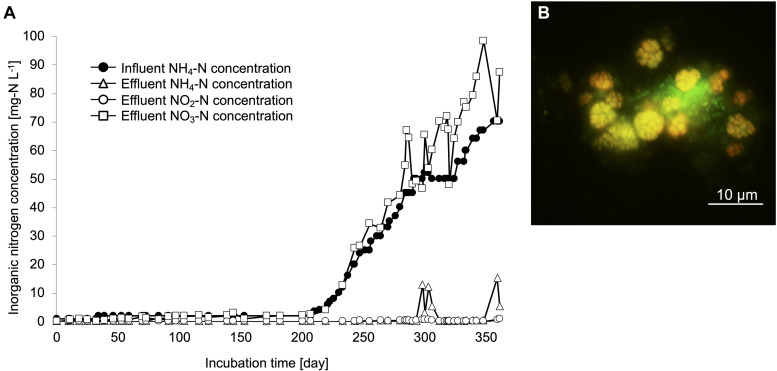
Enrichment culture of nitrifiers in the continuous feeding bioreactor. **(A)** Influent ammonium concentration, and effluent ammonium, nitrite, nitrate concentrations recorded during the incubation period. **(B)** FISH analysis. The yellow cells indicate the Ntspa662-stained *Nitrospira* cells and the green cells indicate the SYTOX green-stained other microorganisms.

Nitrifiers from a drinking water treatment plant were cultivated using zeolites as primary samples from a continuous feeding bioreactor with non-woven fabric materials to maintain the biomass (Supplementary Figure S1). Influent ammonia, effluent ammonia, nitrite, and nitrate concentrations were monitored during the enrichment process (Figure 1A). To increase the biomass in the non-woven fabric materials, zeolites and non-woven fabric materials were set up for an initial 200 days incubation. During this period, ammonia and nitrite concentrations in the bioreactor were maintained at nearly 0 mg-N L^–1^. After 200 days, the influent ammonia concentration was gradually increased from 1 to 70 mg-N L^–1^, in order to enhance the growth activities of the nitrifiers. Even during this period, ammonia and nitrite concentrations were maintained at nearly 0 mg-N L^–1^. Although the ammonia concentration in the bioreactor temporarily increased around the 300 days, the nitrate concentration increased with an increase in the influent ammonia concentration. Therefore, complete nitrification reaction occurred in the bioreactor throughout the enrichment process. FISH observations revealed the growth pattern of nitrifiers in the enrichment samples. Although almost no nitrifiers were identified due to low influent ammonia concentration during the first 200 days, the cells of *Nitrosomonas* and *Nitrospira* species were observed on day 250. Finally, the ratios of species belonging to the *Nitrosomonas* and *Nitrospira* lineage II on day 350 were around 30 and 40%, respectively, based on the cell counts. The species of *Nitrosomonas* and *Nitrospira* that were observed in this study formed spherical and dense microcolonies (Figure 1B and Supplementary Figure S2A). During this enrichment process, AOA and species belonging to *Nitrospira* lineage I were not detected.

**FIGURE 2:**
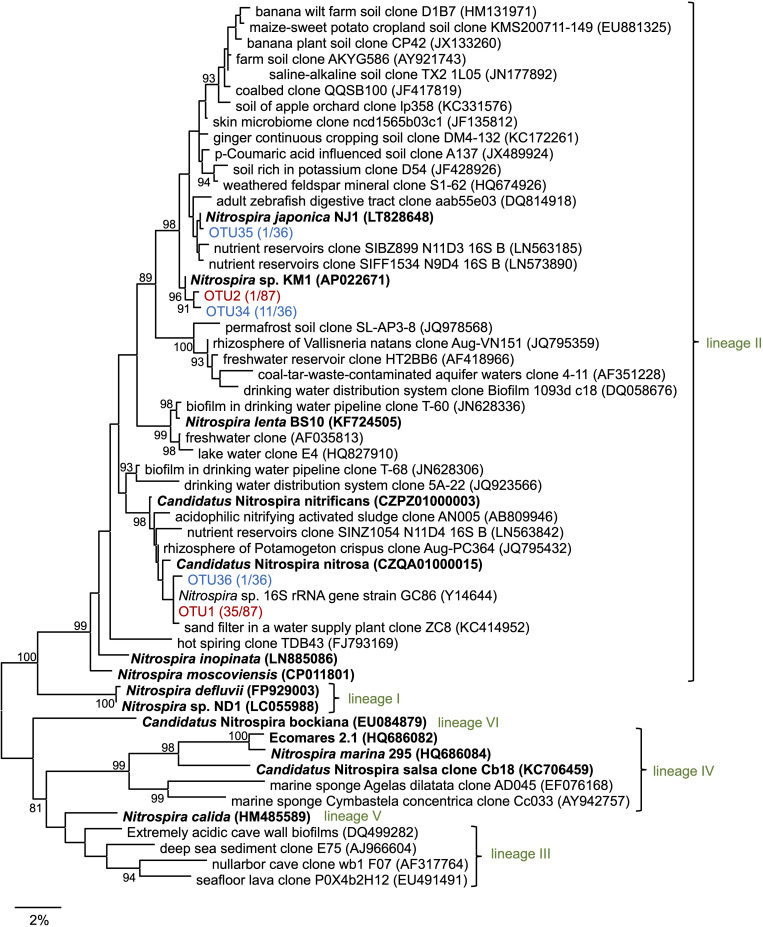
Phylogenetic tree of the genus *Nitrospira* based on the 16S rRNA gene sequence. The tree was constructed using the maximum likelihood (ML) algorithm. Bootstrap values at the branch nodes were iterated 1,000 times. Known isolates and enriched cultures are indicated in bold. For the *Nitrospira* sequences obtained in this study, enrichment clones and isolates are presented in red and blue, respectively. The number of OTUs are specified in brackets. Each lineage belonging to the genus *Nitrospira* is indicated in green. The scale bar corresponds to 2% of the estimated sequence divergence. Accession numbers are shown toward the right side of the organism’s names/descriptions.

Subsequently, 16S rRNA gene sequences in the enrichment culture were cloned on day 350. In total, 87 clones were obtained, out of which 5 and 36 clones were identified to belong to the genus *Nitrosomonas* and *Nitrospira*, respectively (Figure 2, Supplementary Table S2, and Supplementary Figure S2B). The most abundant OTU, OTU1 (35/87 clones) exhibited high sequence similarity with the *Nitrospira* sp. strain GC86 (99.9%). OTU2 (1/87 clones) exhibited high sequence similarity with *Nitrospira japonica* (98.0%). Five clones that were affiliated to the genus *Nitrosomonas* were grouped into three OTUs. Additionally, a total of 48 clones were subjected to sequencing using the primer set for *Nitrospira amoA* gene that was designed in a previous study (Fujitani et al., 2020) and grouped as one OTU (Supplementary Figure S3 and Supplementary Table S2).

### Sorting of Single Microcolonies, Pure Cultivation, and Identification

Enrichment samples were allowed to pass through the cell sorter for sorting single microcolonies according to the method described in the previous studies (Ushiki et al., 2013; Fujitani et al., 2014, 2015; Abe et al., 2017). By sorting an area with a large forward scatter (FSC) value and a small side scatter (SSC) value, we could successfully sort the nitrifiers’ microcolonies (Supplementary Figure S4). Each microcolony was inoculated and cultured in 96 well microtiter plates containing both, ammonia and nitrite inorganic medium. After 1–2 months of incubation period, the cells in each well were subjected to sequencing. In total, 13 isolates that were cultured with the medium containing both ammonia and nitrite belonged to the genus *Nitrospira*. The most abundant OTU, OTU34 (11/13 isolates) exhibited high sequence similarity with *N. japonica* (98%) at the 16S rRNA gene level. OTU35 and OTU36 exhibited over 99% sequence identity with *N. japonica* and *Nitrospira* sp. strain GC86, respectively (Figure 2 and Supplementary Table S2). Additionally, 10 isolates that were cultured with the medium containing both ammonia and nitrite belonged to the genus *Nitrosomonas* (Supplementary Table S2 and Supplementary Figure S2B). Strains that were identified as either *Nitrosomonas* or *Nitrospira* were transferred from the 96 well microtiter plates to test tubes and Erlenmeyer flasks. At that time, the *Nitrosomonas* strains were cultivated using inorganic medium containing only ammonia and the *Nitrospira* strains were cultivated using inorganic medium containing only nitrite. Although some strains might be comammox *Nitrospira*, we identified the organisms with 16S rRNA gene only and could not discriminate comammox *Nitrospira* from nitrite-oxidizing *Nitrospira*. After that, we could not sub-cultivate all the 10 strains that were identified as *Nitrosomonas* in the ammonia-containing medium, and only one strain (OTU34) that was identified as *Nitrospira* grew successfully in the nitrite medium. This successfully cultivated nitrite-oxidizing strain was designated as *Nitrospira* sp. strain KM1. Most of the clones cultivated from the enrichment culture (OTU1, 35 clones) were closely related to *Ca. N. nitrificans* and *Ca. N. nitrosa*, whereas the identified strains (OTU34, 11 clones) were distant from the lineage including *Ca. N. nitrificans* and *Ca. N. nitrosa* (Figure 2). After that, we carefully confirmed the purity of the strain KM1 using heterotrophic media, PCR, FISH.

### Physiological Characterization of Strain KM1

Using scanning electron microscopy, we confirmed that dozens of cells aggregated densely (Supplementary Figure S5A) and that each cell formed a spiral shape (Supplementary Figure S5B). The strain KM1 produced low amounts of extracellular polymeric substances (EPS) (Supplementary Figure S5C) and formed weak flocs in a manner similar to that observed for *Nitrospira lenta*, which also belongs to lineage II of *Nitrospira* (Nowka et al., 2015b). Transmission electron microscopy revealed that the strain KM1 formed a cell cluster embedded in the EPS (Supplementary Figure S5D).

**FIGURE 3:**
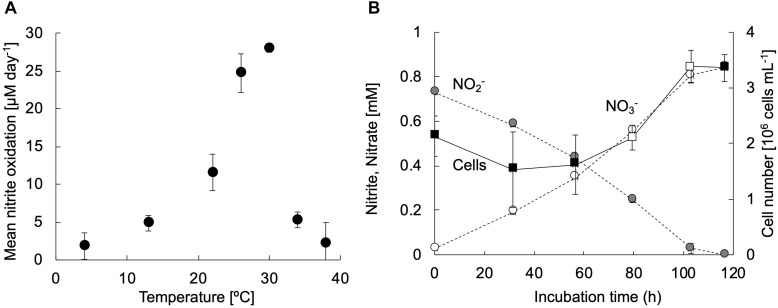
Physiological characteristics of strain KM1. **(A)** The exponentially growing strain KM1 was incubated at a temperature range from 4 to 38°C for 3 days, and the mean nitrite oxidation rates were calculated. **(B)** The strain KM1 was incubated at 28°C in mineral nitrite medium in batch cultures. Gray and white circles indicate the concentrations of nitrite and nitrate, respectively. Filled squares indicate the cell numbers estimated by qPCR analysis targeting the *nxrB* gene. The estimated *nxrB* gene copy number was divided by two, because the *Nitrospira* strain possesses two copies of the *nxrB* gene. The average generation time was determined based on the change in the number of cells in the period between the confirmation of cell growth and the depletion of nitrite. The time points for calculating the minimum generation time were marked by open squares. The experiments were performed in biological triplicate. Error bars indicate the standard deviation (SD).

The strain KM1 showed nitrite oxidation activity over a temperature range from 4 to 38°C and the optimum temperature was identified as 30°C (Figure 3A). This trend was nearly similar those observed for other nitrite-oxidizing *Nitrospira* species (Spieck and Lipski, 2011; Ushiki et al., 2013; Fujitani et al., 2014; Nowka et al., 2015b). To evaluate the generation time and growth yield, the strain KM1 was incubated at 28°C in mineral nitrite medium. It oxidized 0.73 mM of nitrite to nitrate in 117 h. The minimum generation time of the strain KM1 was estimated as 34 h, while the average was estimated as 65 h (Figure 3B and Supplementary Figure S6). Growth yield of the strain KM1 was calculated based on the number of cells and nitrite consumption. The growth yield was estimated as 9.38 log cell mmol NO_2_^–^. Therefore, the growth rate of the strain KM1 was found to be very similar to that of other *Nitrospira* pure cultures (Table 1).

**TABLE 1:** Comparative physiological characteristics among pure *Nitrospira* strains.

**Cultures**	**Lineage**	**Sample source**	***K*_*m*_**	***V*_*max*_**	***K*_*i*__*_*K*_***	**Generation time**	**Growth yield**	**Reference**
			**(μM NO_2_^–^)**	**(μmol NO_2_^–^⋅(mg protein)^–1^⋅h^–1^)**	**(mg-NH_3_ L^–1^)**	**(h)**	**(log cell mmol NO_2_^–^)**	
*Nitrospira defluvii* A17	I	Activated sludge	6 ± 1	48 ± 2	N.D.	37	9.93	a
*Nitrospira* sp. ND1	I	Activated sludge	9 ± 3	45 ± 7	8.5 ± 0.9	14 (74)*	9.61	b
*Nitrospira moscoviensis* M-1	II	Corroded iron pipe	9 ± 3	18 ± 1	N.D.	32	10.55	a
*Nitrospira lenta* BS10	II	Activated sludge	27 ± 11	20 ± 2	N.D.	37	10.40	a
*Nitrospira japonica* NJ1	II	Activated sludge	10 ± 2	31 ± 5	16 ± 2.3	19 (39)*	9.37	b
*Nitrospira* sp. KM1	II	Freshwater	0.81 ± 0.19	2.46 ± 0.33	0.68 ± 0.03	34 (65)*	9.38	In this study
*Nitrospira inopinata* (Comammox)	II	Biofilm on pipe	449.2 ± 65.8	16.88 ± 0.21	N.D.	N.D.	N.D.	c
*Nitrospira* sp. Ecomares 2.1	IV	Aquaculture system	54 ± 11.9	21.4 ± 1.2	N.D.	N. D.	N. D.	d

(a) Nowka et al. (2015a). (b) Ushiki et al. (2017). (c) Kits et al. (2017). (d) Jacob et al. (2017). *Numbers in brackets indicate average generation time.

**FIGURE 4:**
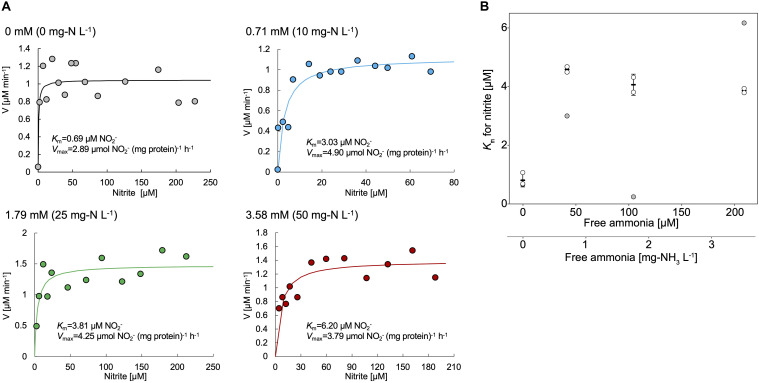
**(A)** Representative data of the kinetic parameters of the strain KM1. Cell pellets of strain KM1 were resuspended in mineral nitrite medium containing different concentrations of NH_4_Cl and were incubated at room temperature with continuous agitation. Circle plots indicate the nitrite consumption in the absence or presence of NH_4_Cl (0 – 3.58 mM). Lines indicate the fitted Michaelis–Menten kinetic equations. **(B)** *K*_*m*_ values for nitrite (μM) plotted against free ammonia concentration (mg-NH_3_ L^–1^ and mM). The experiments were performed using biological triplicates. The values outside the mean ± standard deviation (68.3% confidence interval) of three data in each condition were removed, which are shown as gray circles. The shown mean and standard deviation were recalculated using the remaining plots. All the kinetic data is shown in Supplementary Table S3.

Nitrite oxidation by strain KM1 followed Michalis–Menten kinetics (Figure 4A and Supplementary Table S3), with a mean apparent half-saturation constant *K*_*m*__(app)_ = 0.81 ± 0.19 μM of nitrite and without ammonium. The mean of maximum oxidation rate of nitrite (*V*_*max*_) was 2.46 ± 0.33 μM of nitrite (mg of protein h^–1^). The values of *K*_*m*__(app)_ and *V*_*max*_ were compared among different nitrite-oxidizing *Nitrospira* species (Table 1). Surprisingly, the measured *K*_*m*__(app)_ (NO_2_^–^) and *V*_*max*_ values of strain KM1 were orders of magnitude lower than the published values for other known *Nitrospira* strains. Note that most other *K*_*m*__(app)_ values for NOB were determined by measuring nitrite-dependent oxygen consumption. Although *Nitrospira inopinata* is not strictly a nitrite oxidizer, the genus *Nitrospira* in oligotrophic environments indicated a wide range of affinity for nitrite. Additionally, the values of *K*_*m*__(app)_ and *V*_*max*_ in strain KM1 were compared with other NOB (Supplementary Table S4). Strain KM1 had the lowest value among cultured NOB from non-marine habitats. According to a recent study, *in situ* kinetic values for nitrite oxidation in the dark ocean were at nanomolar levels and orders of magnitude lower than the *K*_*m*__(app)_ values of cultured NOB (Zhang et al., 2020). This would suggest uncultured NOB are well-adapted to oligotrophic environments in nature. NOB in marine samples might have a higher affinity for nitrite than NOB in non-marine habitat. As there is still a gap between *in situ* kinetic values and kinetic values of the cultured representatives, we should continue efforts to characterize uncultured NOB.

Meanwhile, the value of *K*_*m*__(app)_ was shown to increase in the presence of NH_4_Cl, which means that in this condition, the affinities for nitrite decreased in the strain KM1 (Figure 4B). Intriguingly, the strain KM1 seemed to lose the ability to oxidize low concentration of nitrite in the condition where it was exposed to higher concentration of ammonia. The inhibitory constant for free ammonia (*K*_*i*_) for the strain KM1 was calculated as 0.68 ± 0.03 mg-NH_3_ L^–1^ = 40.0 ± 1.89 μM NH_3_, which was lower than that of lineage I *Nitrospira* sp. strain ND1 (8.5 ± 0.9 mg-NH_3_ L^–1^ = 500 ± 52.9 μM NH_3_), and lineage II *N. japonica* strain NJ1 (16 ± 2.3 mg-NH_3_ L^–1^ = 941 ± 135 μM NH_3_) (Ushiki et al., 2017). Additionally, the thermophilic NOB that is related to *Nitrospira calida* showed a tolerance for free ammonia with an IC50 value of 5.0 mg NH_3_-N L^–1^ (Courtens et al., 2016). Therefore, the strain KM1 was more sensitive to ammonia than other strains, such as ND1, NJ1, and thermophilic NOB. Since the *Nitrospira* NXR is located in the periplasm, nitrite transporters most likely play no role in nitrite oxidation (Lücker et al., 2010). Nitrite oxidation by the periplasmic NXR itself seems to be inhibited by ammonia (Courtens et al., 2016; Ushiki et al., 2017), and the mechanism is in need of further study.

The different sensitivity to free ammonia might distinguish the niches of different species of *Nitrospira*. Based on previous microscopic and spatial analysis, *Nitrospira* sp. belonging to lineage I tend to localize in the vicinity of AOB, whereas such close proximity was not observed for the *Nitrospira* sp. belonging to lineage II in nitrifying biofilms (Maixner et al., 2006). This unique distribution was explained based on the small-scale nitrite gradient that forms around the AOB. Furthermore, similar analysis revealed that the preferred nitrite concentrations were different among the members of *Nitrospira* linage I (Gruber-Dorninger et al., 2015). In this context, a gradient of ammonia concentration in the biofilms might be formed depending on the distance from AOB. If ammonia influenced the nitrite affinity of *Nitrospira* species, ammonia seems to be a key factor that influences and regulates niche differentiation among different *Nitrospira* species. Apart from nitrite-oxidizing *Nitrospira*, *N. inopinata*, the only comammox pure culture, possesses a high affinity for ammonia and a high *K*_*m*__(app)_ value for nitrite and therefore appears to be well-adapted to oligotrophic environments (Kits et al., 2017). Considering this point, comammox *Nitrospira* might flourish more than nitrite-oxidizing *Nitrospira* in low ammonia conditions. If AOA and AOB with a high affinity for ammonia coexist with nitrite-oxidizing *Nitrospira*, the competition between comammox *Nitrospira* and nitrite-oxidizing *Nitrospira* seems to maintain the balance in low ammonia conditions. Meanwhile, nitrite-oxidizing *Nitrospira* with high affinity for nitrite would be more abundant than comammox *Nitrospira* in low nitrite and no ammonia conditions. Additionally, continuous feeding with low concentration of ammonia would be effective to enrich comammox *Nitrospira* as demonstrated in our previous study (Fujitani et al., 2020). However, there is a big hurdle to isolate uncultured comammox *Nitrospira*. Although pure microcolonies including comammox *Nitrospira* were probably sorted in this study, we could not confirm cell growth of comammox *Nitrospira*. Considering comammox *Nitrospira* could be adapted to lower oxygen concentrations (Koch et al., 2019), ammonia and oxygen concentration should be strictly controlled in 96 well microtiter plates after sorting. Otherwise, small-scale devices to continuously feed substrate such as microfluidics might be effective to monitor cell growth in single cell levels during long-term incubation.

### Genomic Composition

Genomic properties of the strain KM1 are summarized in Table 2. The sequence coverage was 119.5-fold genome equivalents. The sequence reads obtained from the strain KM1 were assembled into one contig. The reconstructed complete genome sequence of the strain KM1 is 4,509,223 bp in length and with a 56.0% GC content, 4,318 predicted CDS, 46 tRNA, and single copy rRNA genes (5S, 16S, and 23S). The strain KM1 shares high similarity to *N. japonica* strain NJ1 with respect to the 16S rRNA (98.9%), and *nxrB* (93.2%) gene sequences. The genome of strain KM1 contains no *amoA* gene. The ANI between the genome sequences of *Nitrospira* strains ranges from 76.7% to 78.4%. The predicted metabolic features encoded by the genome of strain KM1 are schematically represented in Supplementary Figure S7. We rechecked the purity of strain KM1 from whole genome sequencing data and found no contamination.

**TABLE 2:** Key genomic features of pure strains affiliated with *Nitrospira* lineage II.

**Strain**	***Nitrospira* sp. KM1**	***Nitrospira moscoviensis* M-1**	***Nitrospira japonica* NJ1**	***Nitrospira lenta* BS10**	***Nitrospira inopinata***

**Sample source**	**Drinking water**	**Corroded iron pipe**	**Activated sludge**	**Activated sludge**	**Biofilm on pipe**
Genome size (bp)	4,509,223	4,589,485	4,084,817	3,756,190	3,295,117
GC (%)	56	62	59	58	59
Number of CDSs	4,318	4,863	4,150	3,907	3385
Protein coding density (%)	85.8	90.6	90.3	92.8	89.4
rRNA genes	3	4	3	3	3
tRNA genes	46	47	45	46	48
*nxrA*	3*	5	3	2	1
*nxrB*	2	4**	3	1	1
*nxrC*	3	5***	3	2	4
Nitrogen assimilation	1 (ONR)	1 (ONR)	1 (ONR)	2 (ONR and NirA)	1 (NrfA)
Cyanase	1	1	1	1	0
Urease	1 (UreABCDFG)	1 (UreABCDFGH)	1 (UreABCDEFG)	1 (UreABCDFG)	1 (UreABCDFG)
Hydrogenase	–	1 (group 2a)	–	–	1 (group 3b)
Carbonic anhydrase	1	0	0	1	1
Toxin-antitoxin system	18	50	4	13	16
Reference	In this study	a, b	c	d	e

CDS, coding sequence; ONR, octaheme nitrite reductase. *The length of one of three NxrA (W02_10680) is 4,515 bp, whereas the other NxrA (W02_21980 and W02_23210) are 3,438 and 3,447 bp, respectively. **Two of five *nxrB* genes (NITMOv2_4533 and 4537) are fused after genome deletion event. ***Two of five NxrC (NITMOv2_0740 and 3640) has C-terminal transmembrane helix besides N-terminal signal peptide recognized by the Sec pathway. (a) Koch et al. (2015). (b) Mundinger et al. (2019). (c) Ushiki et al. (2018). (d) Sakoula et al. (2018). (e) Daims et al. (2015).

Like other *Nitrospira* sp., gene sets belonging to the nitrite oxidation pathway and the tricarboxylic acid cycle were identified in the genome sequence of strain KM1. NXR is a key enzyme that catalyzes nitrite oxidation, and is encoded by *nxrA*, *nxrB*, and *nxrC* genes coding for the alpha, beta, and gamma subunits, respectively (Daims et al., 2015). Three copies of potential *nxrA* and *nxrC* genes and two copies of *nxrB* gene were also found in the genome sequence. One of the three *nxrA* genes has a length of 4515 bp, whereas the others are 3437 and 3446 bp in length. A gene likely encoding a sigma-54 dependent transcriptional regulator is directly located in the upstream region of the two *nxrA* genes. Considering the fact that the NXR operons in most *Nitrospira* strains are preceded by sigma-54 (Lücker et al., 2010; Koch et al., 2015), the expression of multicopy *nxrA* and *nxrB* genes might be controlled by the regulator. However, recent transcriptional analysis revealed that the sigma-54 dependent transcriptional regulator exhibited low expression levels during nitrite oxidation in *Nitrospira moscoviensis* (Mundinger et al., 2019).

Nitrite-oxidizing *Nitrospira* utilize nitrite as a major source of nitrogen as well as energy. For reduction of nitrite to ammonia, a gene likely encoding octaheme cytochrome c nitrite reductase (ONR) was found in strain KM1, similar to *Nitrospira moscoviensis* (Koch et al., 2015). NirA-dependent ferredoxin relevant to nitrite reduction was identified in *N. defluvii*, *Nitrospira* sp. ND1 in linage I, and *Nitrospira lenta* in lineage II (Lücker et al., 2010; Koch et al., 2015; Ushiki et al., 2018). Additionally, *Nitrospira inopinata* as a comammox species has a potential *nrfAH* gene, encoding cytochrome c nitrite reductase (Daims et al., 2015). These findings suggest that members of the genus *Nitrospira* possess versatile nitrogen assimilation ability. Genes likely relevant to nitrogen assimilation include *glnA*, glutamine synthetase and *glnK*, and the P-II protein controlling nitrogen assimilation were located close to ONR in the genome of the strain KM1.

In the urease and urea transporter gene clusters, the strain KM1 lacks *ureE* and *ureH* genes, which encode for urease accessory proteins. *UreDEFG* is an accessory protein operon that supports the maturation of urease and controls the activity of urease (Lee et al., 1992). Urease contains Ni in its active center. *UreE* and *UreH* function as a Ni donor and Ni transporter, respectively (Mobley et al., 1995). Although *N. moscoviensis* lacks *ureE* gene, the hydrogenase maturation factors HypA and HypB are expected to exhibit the same function as UreE (Koch et al., 2014, 2015). *Nitrospira* sp. ND1 was shown to perform urease degradation despite the lack of a *ureE* gene, which indicates that urease might be activated with an increase in Ni concentration in the cells because the strain ND1 possesses a gene likely encoding nickel/cobalt transporter with high affinity (Ushiki et al., 2018). However, no chaperon protein and nickel/cobalt transporter with high affinity was identified in the genome sequence of the strain KM1 and may result in low urease activity.

Cyanate hydratase and three copies of an Amt-type ammonium transporter, which may help utilize ammonium as nitrogen source, were also identified in the KM1 genome. Intriguingly, nitrite-oxidizing *Nitrospira* encode three homologs of Amt-type transporters clustering separately in phylogenetic analysis (Koch et al., 2019). If Amt-type transporters among *Nitrospira* are functionally different, *Nitrospira* might possess the potential to survive in environments with fluctuating ammonia concentration. *N. japonica* reduced transcripts of the gene likely encoding octaheme cytochrome *c* nitrite reductase (NSJP_2412) in medium amended by nitrite and ammonium compared to medium with nitrite as a sole nitrogen source (Ushiki et al., 2018). Ammonium addition can also promote growth and allow cultivation of NOB from other genera (Sorokin et al., 2012; Sayavedra-Soto et al., 2015; Ishii et al., 2017; Wegen et al., 2019; Spieck et al., 2020). Therefore, some types of NOB might utilize ammonium from their surroundings instead of assimilatory nitrite reduction. Although it is unknown whether the strain KM1 utilizes ammonium as a nitrogen source, ammonium availability in the environment for NOB would be an important factor.

**FIGURE 5:**
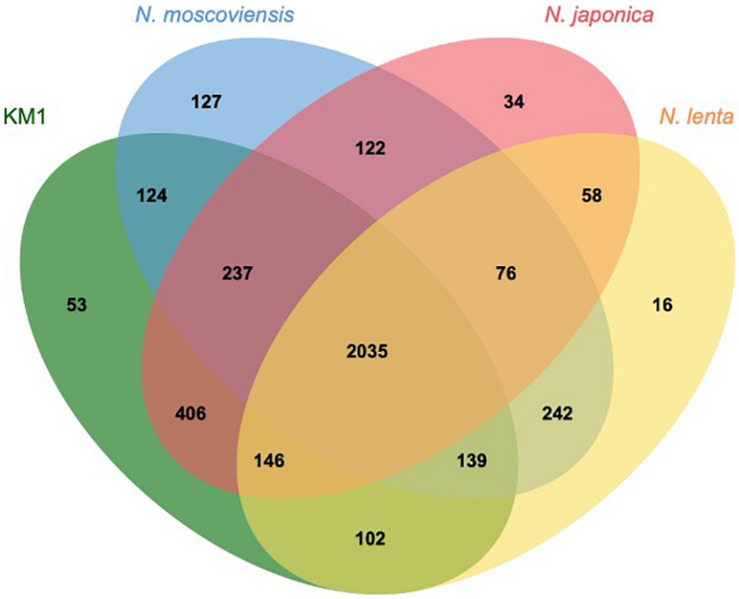
Comparative genomics of isolated *Nitrospira* lineage II. Orthologous genes were clustered by OrthoVenn2 (Xu et al., 2019). The numbers indicate the shared orthologous clusters among the four strains.

Upon comparing the genomes of strain KM1, *N. moscoviensis*, *N. japonica*, and *N. lenta* within *Nitrospira* lineage II, 2035 out of 3917 clusters were shared among all the four strains as orthologous clusters (Figure 5). Carbonic anhydrase (CA) that catalyzes reversible the reaction between carbon dioxide and carbonic acid was identified in strain KM1 and *N. lenta* genome sequences (Sakoula et al., 2018). Additionally, two isolated *Nitrospira* strains in lineage I have CA (Lücker et al., 2010; Ushiki et al., 2018). According to previous studies, the growth of *Haemophilus influenza* lacking CA was inhibited by atmospheric concentrations (∼0.04% CO_2_), which indicates that CA plays an important role in CO_2_ poor conditions (Langereis et al., 2013). Considering that CA is essential for survival in many microorganisms (Kusian et al., 2002; Aguilera et al., 2005; Burghout et al., 2011), CA might be one of the factors that could facilitate ecological niche differentiation among different *Nitrospira* species. Additionally, 18 sets of genes likely encoding for a toxin-antitoxin (TA) system in a stress response module (Williams and Hergenrother, 2012) were identified in the genome of strain KM1. In *Nitrosomonas europaea*, a toxin endoribonuclease designated as MazF_*NE*__1181_ may improve a resistance to mercury in heavy metal stress environments (Miyamoto et al., 2016). This suggests that strain KM1 might respond to different environmental stresses using its TA system.

## Conclusion

This study reported the isolation, morphology, physiology, kinetics, and genome of a novel nitrite-oxidizing *Nitrospira* strain from a fixed-bed column present at a drinking water treatment plant. Although morphological and genomic features were typical of *Nitrospira*, the values of *K*_*m*__(app)_ and *V*_*max*_ for nitrite of the strain KM1were orders of magnitude lower than the cultured NOB in non-marine habitat. Therefore, the strain KM1 is well-adapted to oligotrophic environments with a high affinity for nitrite. To bridge the gap between *in situ* kinetic values and kinetic values of the cultured representatives, further studies including the isolation and characterization of novel NOB strains would be required. Additionally, strain KM1 had high sensitivity to free ammonia. Considering ammonia influenced *K*_*m*__(app)_ for nitrite, ammonia as well as nitrite concentration could be the main determinants for niche differentiation among NOB. These findings broaden known physiological features of nitrite-oxidizing *Nitrospira*, an ecologically important group in the biogeochemical nitrogen cycle.

## Data Availability Statement

The National Center for Biotechnology Information (NCBI) BioProject number for genome sequencing of *Nitrospira* sp. strain KM1 is PRJDB5488 (https://www.ncbi.nlm.nih.gov/bioproject/608100). Illumina raw reads were deposited at the DDBJ Sequence Read Archive (SRA) under the accession number DRR084142 (https://trace.ncbi.nlm.nih.gov/Traces/sra/?run=DR R084142). The reconstructed genome sequence of the strain KM1 is deposited in the NCBI under the accession number AP022671 (https://www.ncbi.nlm.nih.gov/nuccore/AP022671.1/). Sequences obtained by cloning are also available in the NCBI under the accession numbers LC521604 – LC521648.

## Author Contributions

HF and ST contributed to the conception and design of the study. HF and KM sampled out the zeolite from the drinking water treatment plant and enriched the nitrifiers. KM and NU performed the isolation of nitrifiers and cloning based on 16S rRNA gene. MN and KI carried out the cloning based on *amoA* gene. KI conducted the physiological experiments. HF and KI analyzed the physiological data. YS performed the genome sequencing and assembly. HF, KI, and SK carried out the genome analysis. HF, KI, YS, and ST wrote the manuscript. All authors contributed to the article and approved the submitted version.

## Conflict of Interest

The authors declare that the research was conducted in the absence of any commercial or financial relationships that could be construed as a potential conflict of interest.
