# Supplementary material for: Genomic and Physiological Characteristics of a Novel Nitrite-Oxidizing Nitrospira Strain Isolated From a Drinking Water Treatment Plant
Source: Front Microbiol. 2020 Sep 15;11:545190. doi: 10.3389/fmicb.2020.545190 (PMC7522533; doi:10.3389/fmicb.2020.545190)
Supplement: Supplementary file 1 [file Data_Sheet_1.pdf]

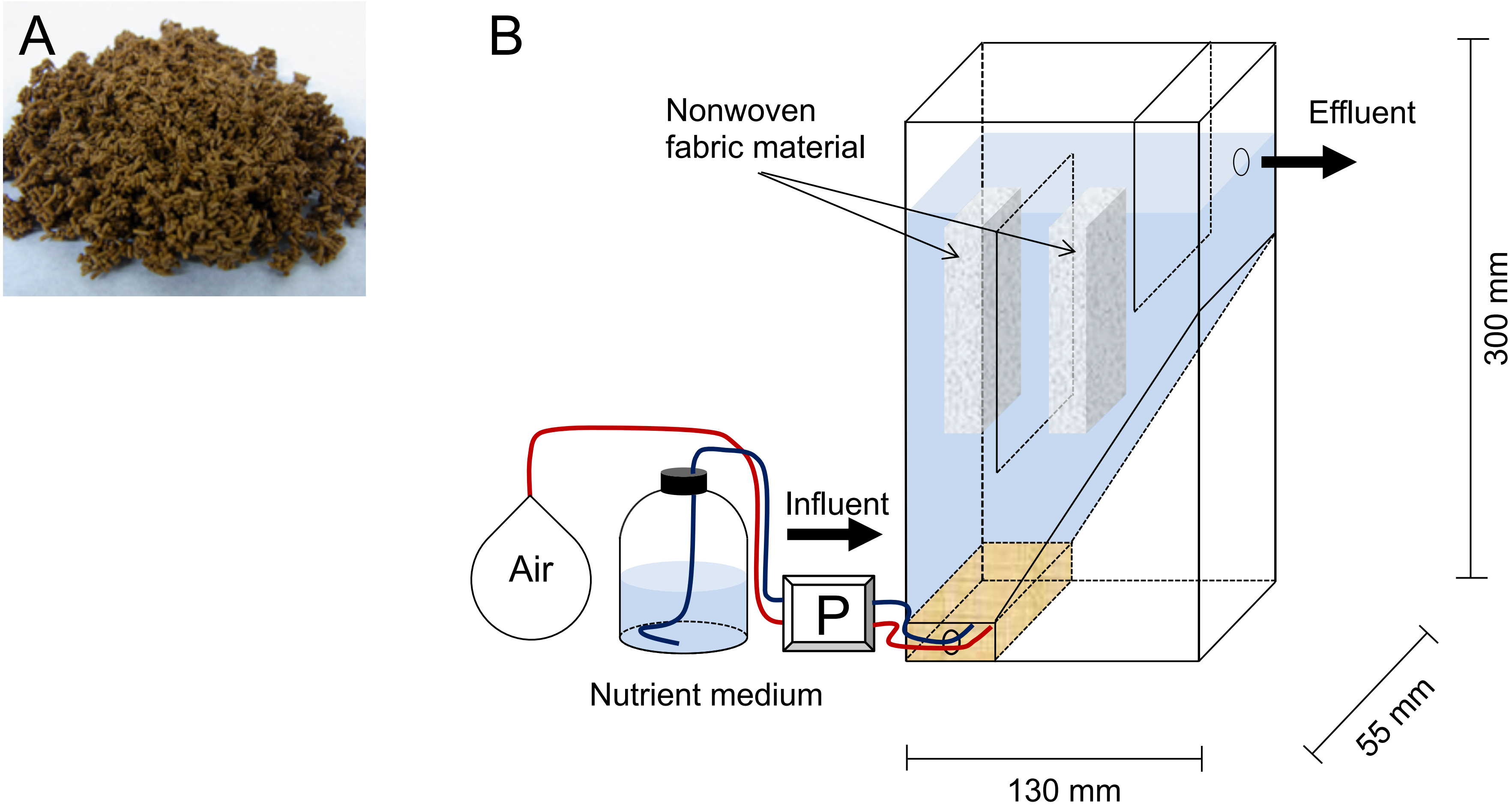

Supplementary figure S1. Enrichment of nitrifiers using a continuous feeding bioreactor.
(A) Zeolite as water purification materials set up from a fixed-bed column in a drinking water treatment plant.
(B) Schematic draw of a continuous feeding bioreactor to enrich nitrifiers.

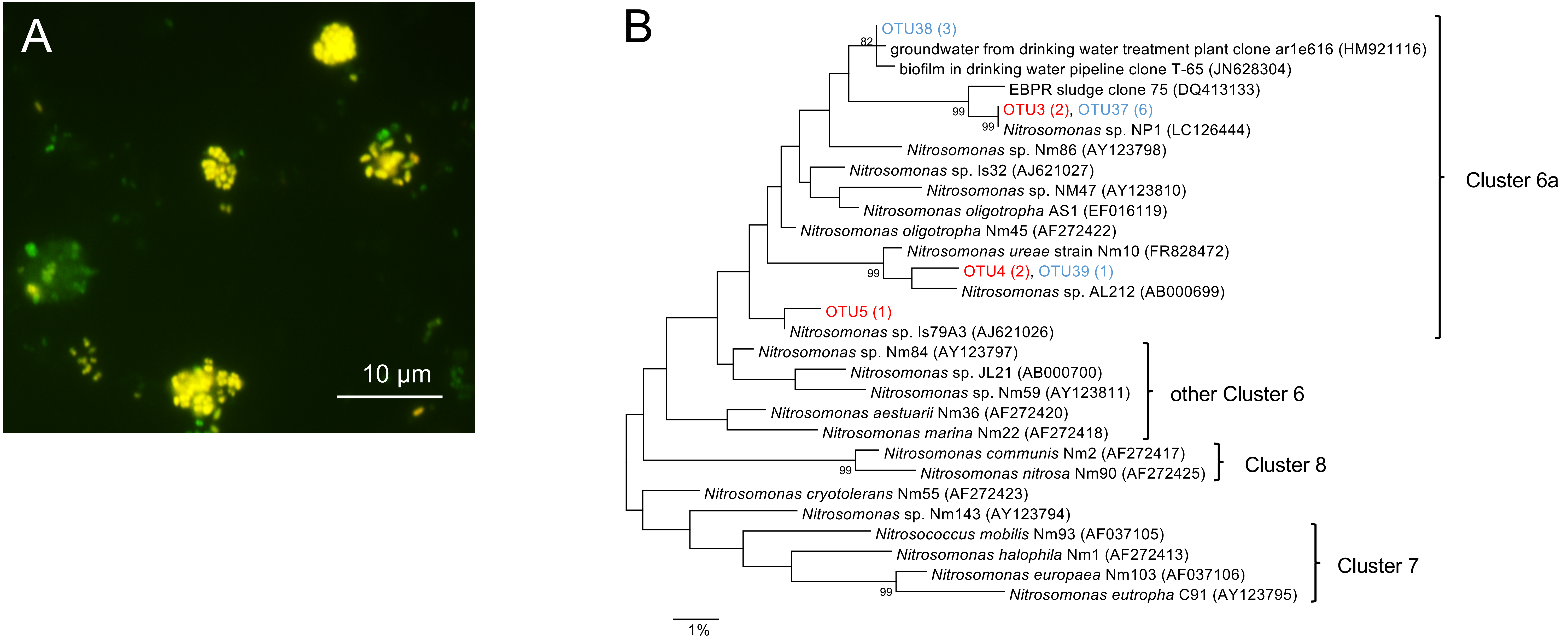

Supplementary figure S2. *Nitrosomonas*-like bacteria enriched in a continuous feeding bioreactor.
(A) FISH image of *Nitrosomonas* cells. Enrichment samples on day 350 were observed in FISH. Nso190 probe labeled with Cy3 was used to detect *Nitrosomonas* cells. The yellow cells are Nso190-stained *Nitrosomonas* cells. Green cells are SYTOX green-stained other microorganisms.
(B) Phylogenetic tree of the genus *Nitrosomonas* based on 16S rRNA gene sequence. The tree was constructed using the maximum likelihood algorithm. Bootstrap values at the branch nodes were iterated 1,000 times. Enriched cultures and isolates obtained in this study are shown in red and blue, respectively. The number of OTUs are noted in brackets. The scale bar corresponds to 1% estimated sequence divergence. Accession numbers are shown to the right of the organism names/descriptions.

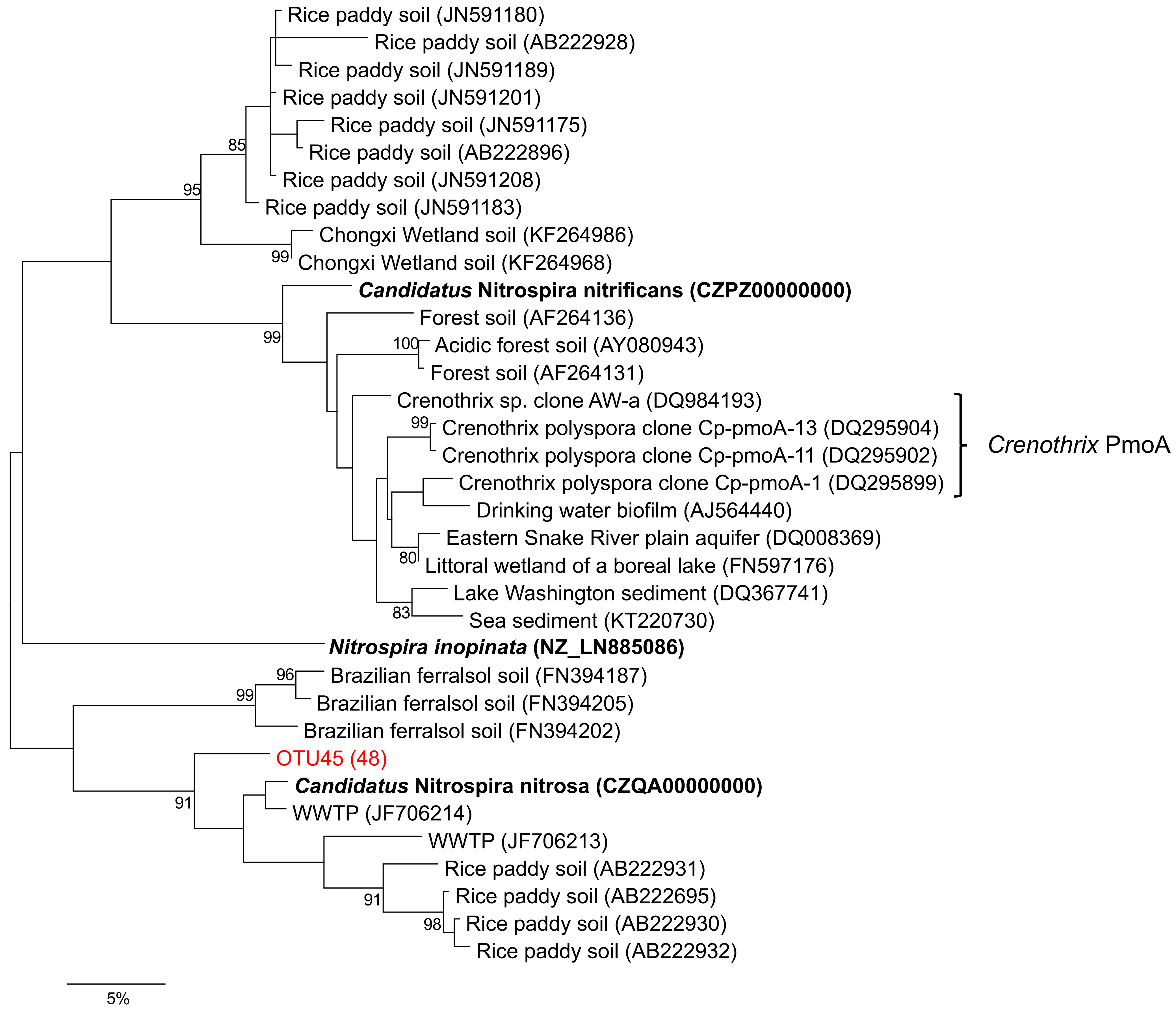

Supplementary figure S3. Phylogenetic tree based on comammox *amoA* gene sequence.
The tree was constructed using the maximum likelihood algorithm. Bootstrap values at the branch nodes were iterated 1,000 times. Known isolates and enriched cultures are in bold. Enrichment clone obtained in this study is shown in red. The number of OTUs are noted in brackets. The scale bar corresponds to 5% estimated sequence divergence. Accession numbers are shown to the right of the organism names/descriptions.

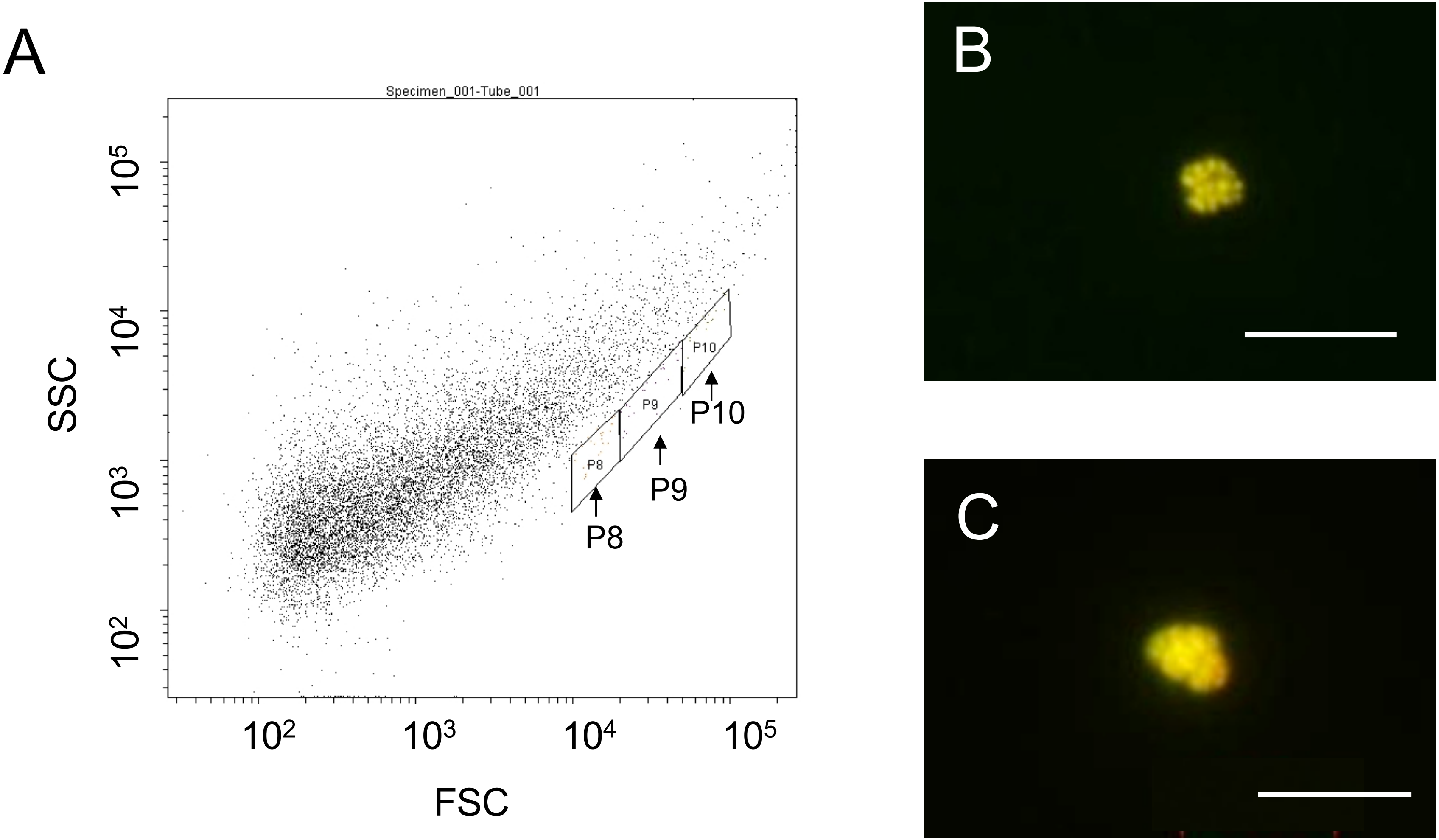

Supplementary figure S4. Separation of microcolonies by a cell sorter.

(A) Dot plot generated based on FSC and SSC. Focused on areas with larger FSC value and smaller SSC value (P8, P9, and P10), the area including high abundance of nitrifiers' microcolonies were explored. (B) A microcolony of Nso190-stained *Nitrosomonas* cells sorted from P8 (C) A microcolony of Ntspa662-stained *Nitrospira* cells sorted from P8. Scale Bar is 10 μm.

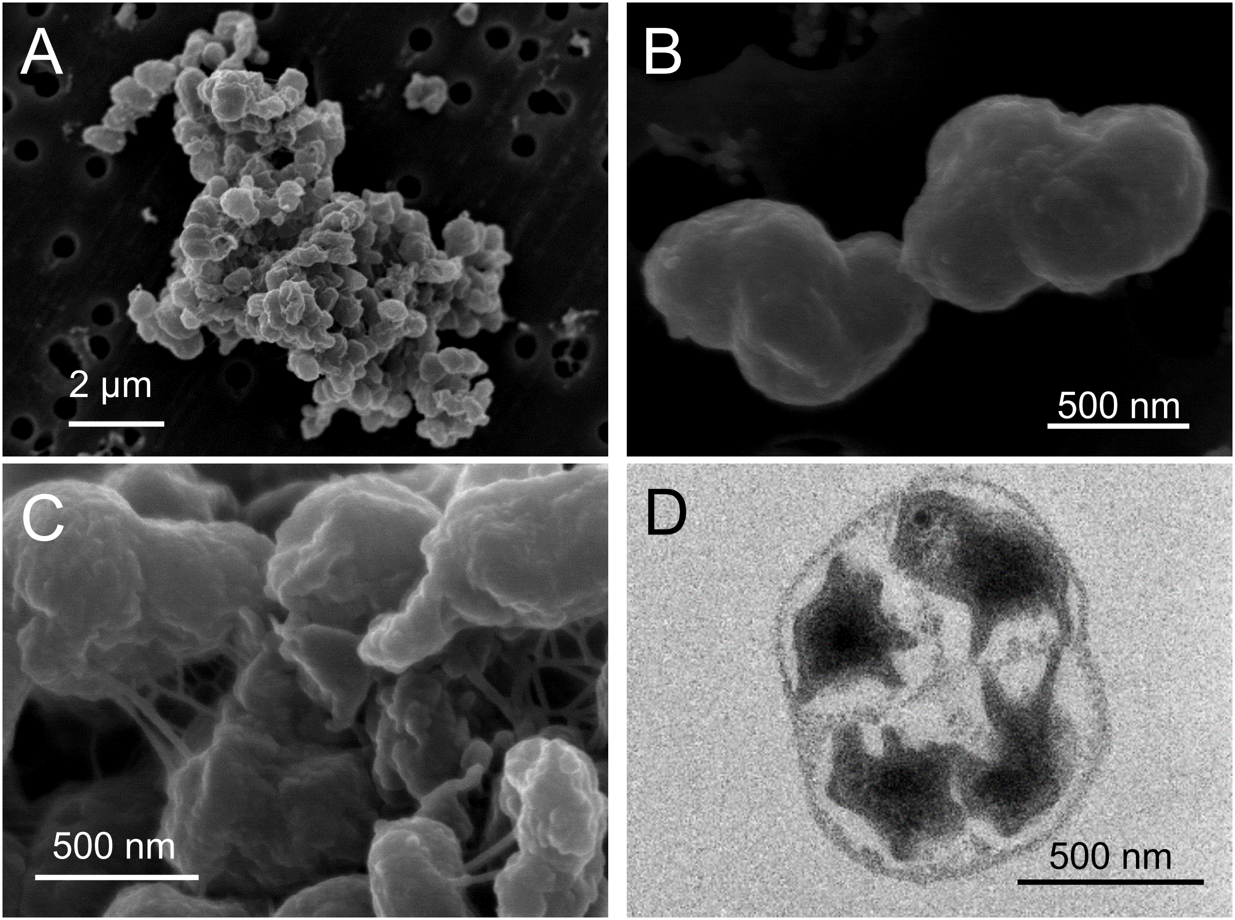

Supplementary figure S5. Morphology of the strain KM1. Scanning electron microscopic images of (A) a microcolony, (B) single cells (C) a magnified view within a microcolony. (D) Transmission electron microscopic image depicting the ultrathin section of a microcolony.

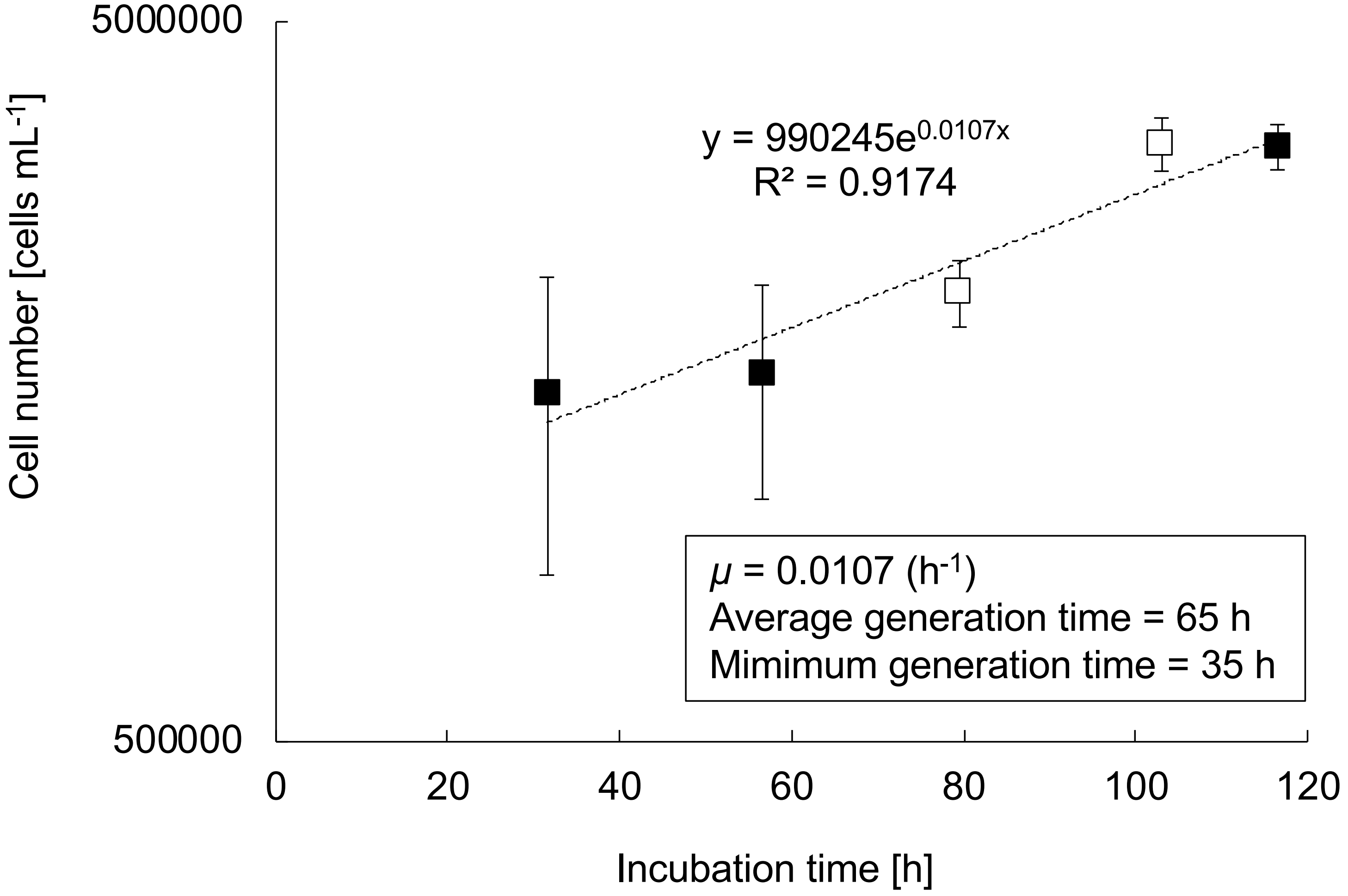

Supplementary figure S6. The log-transformed cell number in the exponential growth phase.

The growth rate (μ) and the average generation time (G_t) were calculated the following equation: $G_t = \ln(2) / \mu$. The minimum generation time was calculated using the KM1 cells in the log phase between the two time points (open squares). The whole growth curve includes the lag and stationary phase. The experiments were performed in biological triplicate. Error bars indicate the standard deviation. Figure S6 is produced from **Figure 4** in the main text.

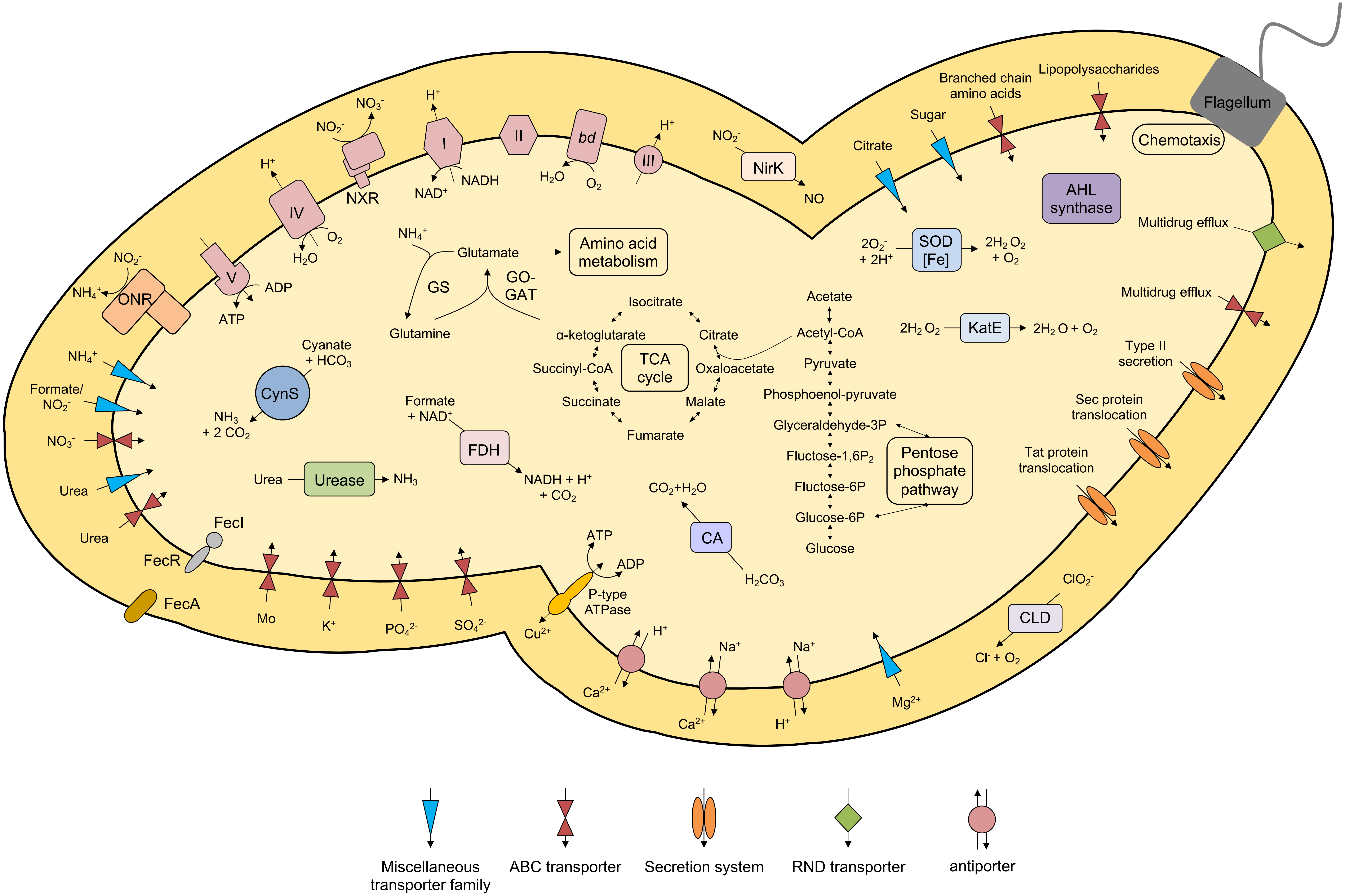

Supplementary figure S7. Schematic depicting the metabolic pathways of the strain KM1 based on the genes annotated by DFAST (Tanizawa *et al.*, 2018).

Abbreviations: CA, carbonic anhydrase; CLD, chlorite dismutase; CynS, cyanate hydratase; FDH, formate dehydrogenase; FecA, Ferric citrate outer membrane transporter; FecIR, Ferric citrate related regulatory proteins; GS, glutamine synthetase; GOGAT, glutamate synthase; KatE, catalase; NirK, copper-containing nitrite reductase; NXR, nitrite oxidoreductase; ONR, octahaeme cytochrome c nitrite reductase; SOD [Fe], Superoxide dismutase [Fe]; TCA cycle, tricarboxylic acid cycle.
